# Prevalence and determinants of overweight or obesity among medical students over a 2-year observation

**DOI:** 10.3389/fnut.2024.1437292

**Published:** 2024-08-21

**Authors:** Szymon Szemik, Iwona Zieleń-Zynek, Ewa Szklarek, Małgorzata Kowalska

**Affiliations:** ^1^Department of Epidemiology, Faculty of Medical Sciences in Katowice, Medical University of Silesia in Katowice, Katowice, Poland; ^2^Students Scientific Association of the Department of Epidemiology, Faculty of Medical Sciences in Katowice, Medical University of Silesia in Katowice, Katowice, Poland

**Keywords:** overweight, obesity, public health policy, medical students, prospective study

## Abstract

**Introduction:**

University students are a special population group characterized by changes in BMI values over the subsequent years of education, with an upward tendency to BMI. The presented study aims to evaluate the prevalence of overweight and obesity and their determinants in medical students during the 2-year follow-up observation.

**Materials and methods:**

We analyzed data collected from the first follow-up of the cohort study named “POLLEK” conducted among medical students at the Medical University of Silesia in Katowice. Students were followed at two points of time: in their inaugural year of studies (the academic year 2021/2022, T1, *N* = 427), and subsequently in their second year (the academic year 2022/2023, T2, *N* = 335).

**Results:**

In the initial year of evaluation, 371 individuals (86.9%) exhibited normal body weight, 47 (11.0%) were overweight, and 9 (2.1%) were classified as obese. Subsequent assessments during the second year revealed the following distribution: 277 students (84.2%) with normal body weight, 40 (12.2%) classified as overweight, and 12 (3.6%) identified as obese. In summary, regardless of the academic year, an increased risk of being overweight or obese was significantly associated with dissatisfaction with personal health, financial strain, and a diet abundant in animal products.

**Conclusion:**

The results of our study confirmed an increase in the prevalence of overweight or obesity among medical students during the 2-year follow-up observation. Significant determinants of overweight or obesity among medical students were: dissatisfaction with individual health status, male sex, financial deficiencies, and a diet abundant in meat consumption.

## 1 Introduction

According to the World Health Organization (WHO) reports, more than 20% of people in the European region are obese. Obesity, diagnosed with a Body Mass Index (BMI) value above 29.9 kg/m^2^, is recognized as the most dangerous chronic disease, which—untreated—could lead to hormonal disorders, type 2 diabetes, metabolic syndrome, development of cardiovascular diseases, and finally, increases the risk of cancers (1, 2). Numerous scientific studies show that university students are a special population group characterized by changes in BMI values over the subsequent years of education, with an upward tendency to BMI. There are many determinants in the academic life of freshman university students that can influence BMI changes in subsequent years of study, including eating habits, physical activity, alcohol consumption, and stress levels (3, 4). Changes in BMI observed among students over time may predispose to changes in the quality of life (QoL) of these students, both in the present and later years of their lives. Quality of life (QoL) is “an individual’s perception of their position in life in the context of the culture and value systems in which they live and in relation to their goals, expectations, standards, and concerns” (5–7).

Medical students are a social group that should be characterized as sustaining possibly the highest quality of life to maintain motivation to learn. QoL assessment among medical students is used to identify the range of problems this group struggles with, which may affect their later professional life. QoL can be influenced by many factors, including stress level, eating habits, stimulant use, sleep, and weight disorders. Scientific researchers have found that chronic stress associated with medical studies promotes eating disorders and alcohol or drug abuse (8). Incorrect eating habits predispose to poor nutritional status. Researchers have observed a correlation between a high level of stress and nutritional status, assessed by anthropometric indicators such as Body Mass Index (BMI). BMI is a useful screening tool to evaluate nutritional status, which is an easy, non-invasive, cheap method of assessing body weight (9). Both overweight and undernutrition among university students can have a significant impact on their academic quality of life, including concentration, sleep, memory, and physical and intellectual condition. Medical students’ life patterns are somewhat different from university students of other facilities in the same age group. Following Carl Rogers’ humanistic theory, people throughout their lives undergo a process of change correlated with self-development. Medical students’ ability to learn and adapt to changes plays a key role in the process of creating their QoL (10, 11).

Overweight and obesity are increasing worldwide—in children, adolescents, and adults. World Health Organization’s statistics predict that global obesity prevalence is going to reach 21% in women and about 18% in men by 2025. Excessive body weight is perceived as one of the most serious public health problems in Poland. University years have had a high influence on shaping adult habits, concerning everyday diet, physical activity, tobacco smoking, and alcohol drinking. Acquiring unhealthy lifestyle behaviors at an early stage of life may also result in a distorted body view. Therefore, it is very important to investigate body weight changes in students during their university education (12–15).

Early detection of weight disorders among students, especially medical students can be used to plan strategies to improve their future quality of life and prevent the occurrence of obesity-related chronic diseases in the future.

Considering the above, the presented study aims to evaluate the prevalence of overweight and obesity and their determinants in medical students during the 2-year follow-up observation.

## 2 Materials and methods

### 2.1 Study design

The data presented in this paper was collected in a follow-up cohort study called POLLEK (POLski LEKarz), undertaken among medical students at the Medical University of Silesia in Katowice. Our earlier primary papers have delved into analyses concerning their quality of life, general health levels, and patterns of alcohol consumption, encompassing even the period of the COVID-19 pandemic (13, 16, 17). We have included all students in the study who entered the medical school in the academic year 2021/2022. The questionnaire surveys were conducted at two distinct time points, initially during their inaugural year of studies (the academic year 2021/2022), and subsequently during their second year (the academic year 2022/2023). The total number of participants during the initial period was 427, reflecting an 83.7% response rate. In contrast, during the subsequent period, the participant count was slightly lower at 335, maintaining a consistent response rate of 83.8%. During the observation period, the dropout rate was determined at 21.5%, primarily attributed to students withdrawing from studies (*n* = 87) or failing to attend on the measurement day (*n* = 5). Data collection was facilitated through paper-form questionnaires, with written informed consent obtained from all participants. Each participant was assigned a unique, anonymous identifier (ID), which was also included on the distributed paper forms. This implementation of IDs aimed to facilitate follow-up procedures in subsequent phases of the study while ensuring complete anonymity. The study received approval from the Bioethics Committee of the Medical University of Silesia in Katowice (approval number KNW/0022/KB/217/19; dated 8 November 2019).

### 2.2 Measures

The POLLEK questionnaire encompassed a set of questions, including assessments related to Quality of Life (QoL), the incidence of hazardous alcohol consumption, sociodemographic factors (such as sex, age, current financial status, and residence during medical studies), lifestyle indicators (including current usage of traditional or electronic cigarettes, and frequency of physical activity), as well as health-related variables (self-reported health status, presence of chronic illnesses, measurements of body weight and height for BMI calculation, and systolic and diastolic blood pressure indices). Measurements of systolic (SBP) and diastolic (DBP) blood pressure were conducted after a min. of 5-min rest in the sitting position. The cuffs of the blood pressure monitors were adapted to the dimensions of the arm. Body weight and height measurements were carried out during the whole study period using the same validated device (Body Composition Analyzer Model: ioi 353 manufactured zby Jawon Medical Co., Ltd.), and under the supervision of a research team member.

To identify the overweight and obesity among study participants, the criteria of the World Health Organization (WHO) have been used, which are as follows:

- overweight as a BMI greater than or equal to 25; and
- obesity as a BMI greater than or equal to 30 (18).

Then, for statistical analysis, two categories of blood pressure were established:

- normal: systolic less than 120 mm Hg; diastolic less than 80 mm Hg (19); and
- high: systolic greater than or equal to 120 mm Hg; diastolic systolic greater than or equal to 80 mm Hg.

Quality of Life (QoL) was assessed across four domains (physical health, psychological, social relationships, and environmental domain) using the WHOQOL-BREF questionnaire (20), validated in Polish. Respondents rated aspects of their QoL as satisfactory or not, transformed into a 0–100 scale per WHO guidelines. Approval for using the questionnaire in the POLLEK study was obtained from WHO.

The prevalence of hazardous alcohol consumption among medical students was evaluated using a Polish version of the AUDIT questionnaire, developed by WHO in 1989. This 10-item tool screens for excessive alcohol consumption, covering recent use, dependence symptoms, and related issues. Each item scores from 0 to 4, with a maximum of 40 points. Hazardous drinking was identified with a score of ≥8 points (13).

### 2.3 Dietary assessment

Students’ dietary habits were evaluated by using a qualitative assessment method developed by Starzyńska. The questionnaire contains six items identifying the number of meals per during the day planned in the menu (4–5, 3, less than 3), and frequency of consumption of individual nutrients such as Nowicki et al. (21) and Mazurek-Kusiak et al. (22):

- the number of meals with animal protein (in all meals vs. 75% of meals vs. fewer meals),
- frequency of milk or cheese consumption (daily in at least two meals, as opposed to daily in at least one meal and 50% of days in two meals vs. less often);
- frequency of vegetables or fruits (daily in at least three meals, as opposed to daily in at least two meals vs. less often);
- frequency of raw vegetables or fruits (daily vs. 75% of meals vs. less often); and
- frequency of wholemeal bread, groats, and dry leguminous vegetable consumption (daily at least in one meal, in 75% of meals, or at least one of the products mentioned, less often).

In each question, the respondents could receive 5 points, 3 points, or 0 points resulting in a possible score range between 0 and 30 points. The total points represent the dietary assessment within the categories such as a good diet with 30 points when eating well; adequate diet with 21–28 points when eating adequately; almost adequate diet with 12–20 points but without 0 scores; 11 points and below was characterized as poor diet (21).

### 2.4 Statistical analysis

The qualitative variables were presented by the number of observations and frequency. The quantitative variables were described as mean values and standard deviations. Due to the Shapiro–Wilk test results, all statistical procedures have been performed using nonparametric analyses. Consequently, a Spearman correlation has been used to assess the relationship between quantitative variables. Moreover, the Wilcoxon signed-rank test for paired quantitative variables was adopted to assess differences between both years of observation. Additionally, McNemar’s test has been used to assess the same differences in paired nominal data. Differences between groups specified in T1 and T2 were tested by the U-Mann Whitney test for the continuous variables and the chi-squared test for categorical variables. Finally, odds ratio values have been calculated to estimate the relationship between categorial dependent and explanatory variables. The level of statistical significance in all analyses was considered at *p* values below 0.05. All statistical analyses were undertaken using the Statistica 13.3 package (TIBCO Software Inc., Palo Alto, CA, United States).

## 3 Results

The presented study involved 289 female and 138 male first-year students (67.7 and 32.2% of the study group respectively). In the second year, the study was continued among 226 female (67.5%) and 109 male students (37.5%). The comparison of medical students in their first and second years of observation regarding selected quantitative variables is presented in Table 1. In terms of BMI calculation, a significant increase in score has been identified during the second year of observation. Additionally, it should be noted that values indicating systolic and diastolic pressure have also increased during the same period. Simultaneously, the QoL scores of students decreased in their second year of studies in the somatic and psychological domains.

**Table 1:** The comparison of medical students in their first and second years of observation concerning selected quantitative variables.

Variable	The first year of observation (*N* = 427)		The second year of observation (*N* = 335)		*p* -value
	M	SD	M	SD	
Age	19.96	1.76	20.80	1.57	<0.001
Weight	64.16	13.53	65.32	14.35	<0.001
Height	172	10.0	171	10.0	<0.001
BMI	21.64	3.26	22.11	4.12	<0.001
Systolic blood pressure	114.44	12.32	117.59	11.79	<0.001
Diastolic blood pressure	72.41	8.47	76.11	11.17	<0.001
QoL somatic domain	62.82	15.15	44.96	12.25	<0.001
QoL psychological domain	62.23	16.80	49.9	12.0	<0.001
QoL social relationship domain	69.51	18.57	69.34	19.15	0.379
QoL environmental domain	64.35	12.57	66.18	13.59	0.001
AUDIT score	6.06	4.49	6.03	4.11	0.621

M, Mean; SD, Standard deviation; QoL, Quality of life; *p*, Statistical significance.

Table 2 shows results of dietary habits assessment in medical students, in both years of observation. It has been observed that the proportion of students who declared a poor dietary assessment was relatively higher during the second year of an investigation (37.2 vs. 32.9% in the first year). Conversely, the percentages of students categorized as having almost adequate or adequate/good dietary assessments were slightly lower in the second year compared to the first.

**Table 2:** Percentage and number of medical students in particular dietary habits assessment group in the first and second years of observation according to obtained scoring in Starzyńska questionnaire.

Year of observation	Dietary assessment							
	Poor diet		Almost adequate diet		Adequate/Good diet		Missing data	
	*N*	%	*N*	%	*N*	%	*N*	%
First (*N* = 427)	139	32.6	212	49.6	71	16.6	5	1.2
Second (*N* = 335)	124	37.0	159	47.5	50	14.9	2	0.6

In the next stage, descriptive statistics have been provided for the selected qualitative variables across both observation periods, with differences being verified through statistical significance tests (Table 3). Notably, students examined during the second year of observation exhibited a significantly higher frequency of living outside the family home compared to the first year (*p* = 0.016). Moreover, they declared significantly lower rates of current tobacco smoking (<0.001). Concurrently, a higher incidence of chronic disease diagnosis was observed (*p* = 0.014), alongside a decreased consumption of wholemeal bread, groats, and dry leguminous vegetables in their daily diet (*p* = 0.008).

**Table 3:** Differences in the number and the percentage across categories defined by selected explanatory variables—data for both observational years.

Variable		T1		T2		*p*-value
		*N*	%	*N*	%	
Current place of residence during studies at university	Family home	124	29.0	87	26.0	0.016
	Dormitory/rented flat or room	303	71.0	247	74.0	
Current cigarette smoking (traditional or electronic)	Never or in the past, not now	358	84.1	296	88.6	<0.001
	Yes, still	68	15.9	38	11.4	
Ever diagnosed with chronic disease	Yes	101	23.8	95	28.4	0.014
	No	324	76.2	240	71.6	
Frequency of wholemeal bread, groats, and dry leguminous vegetables consumption	Daily at least in one meal, at least 75% of meals	273	64.1	187	55.8	0.008
	Less often	153	35.9	148	44.2	

N, Number; *p*, statistical significance; T1, The first year of study; T2, The second year of study.

Then, the differences between BMI, and blood pressure values in the groups of students were defined by selected qualitative variables identifying elements of lifestyle, dietary behaviors, or financial situation across both years of observation (Table 4). In the initial year of medical university, significantly higher BMI values concerned students declaring a worse financial situation (*p* = 0.034) and those indicating a worse health status (*p* = 0.042). However, these discrepancies dissipated by the second year. On the other hand, during the second year, significantly higher BMI values were linked to students who did not or rarely engaged in physical activity (*p* = 0.046) and consumed more fruit and vegetables in their daily diet (*p* = 0.048). Furthermore, it is noteworthy that consistently higher BMI values were evident among students consuming animal protein daily, compared to those who consumed it less frequently, and this observation persisted throughout both years of the study.

**Table 4:** Differences in BMI and blood pressure values in the groups were defined by selected qualitative variables identifying elements of lifestyle, dietary behaviors, or financial situation in both years of observation.

Explanatory variable		BMI					
		T1		*p*-value	T2		*p*-value
		M	SD		M	SD	
Current financial situation	Average or poor	22.3	3.7	0.034	22.4	4.3	0.230
	Good or very good	21.4	4.0		22.0	3.7	
Frequency of physical activity	Yes, regularly or less often	21.5	3.1	0.374	21.6	5.3	0.046
	Not at all	22.1	3.9		22.2	3.7	
Current health status	Average or poor	22.1	2.9	0.042	22.2	4.0	0.679
	Good or very good	21.3	3.6		20.0	4.2	
Number of meals containing animal protein	In all meals, at least 75% of meals	22.1	3.4	<0.001	22.8	4.5	<0.001
	Fewer meals	20.9	2.8		21.1	3.2	
Frequency of vegetable or fruit consumption	Daily in at least twice a day	21.5	3.1	0.264	22.3	3.7	0.048
	Less often	21.9	3.6		21.8	4.8	
**Explanatory variable**		**Systolic blood pressure**					
		**T1**		***p***-value	**T2**		***p***-value
		**M**	**SD**		**M**	**SD**	
Declared quality of life	Average or poor	112.5	11.3	0.068	116.6	13.6	0.552
	Good or very good	115.1	12.6		117.7	11.0	
Satisfaction of health status	Yes, satisfied	112.1	11.7	0.099	116.0	12.8	0.444
	Average or dissatisfied	115.2	12.6		118.2	11.1	
Frequency of alcohol consumption	Never or rarely	114.4	12.3	0.961	116.9	12.1	0.085
	More often than 2–4 times a week	114.5	12.4		122.8	7.5	
Frequency of physical activity	Yes, regularly or less often	113.7	13.6	0.641	116.5	12.5	0.062
	Not at all	114.6	12.0		121.2	8.2	
Current health status	Average or poor	113.8	12.1	0.353	115.1	13.1	0.056
	Good or very good	114.9	12.5		119.1	10.7	
Number of meals containing animal protein	In all meals, at least 75% of meals	116.9	12.8	<0.001	119.3	10.7	0.086
	Fewer meals	111.2	10.9		115.5	12.8	
Frequency of wholemeal bread, groats, and dry leguminous vegetables consumption	Daily at least in 1 meal, at least 75% of meals	113.6	12.1	0.097	117.2	12.8	0.907
	Less often	115.9	12.7		118.1	10.3	
**Explanatory variable**		**Diastolic blood pressure**					
		**T1**		***p***-value	**T2**		***p***-value
		**M**	**SD**		**M**	**SD**	
Satisfaction of health status	Yes, satisfied	72.7	8.3	0.117	77.7	12.9	0.024
	Average or dissatisfied	71.8	8.8		72.6	9.7	
Frequency of physical activity	Yes, regularly or less often	72.3	8.4	0.845	75.4	12.2	0.051
	Not at all	73.0	9.0		78.2	6.4	
Number of meals containing animal protein	In all meals, at least 75% of meals	73.0	8.7	0.048	76.6	12.3	0.497
	Fewer meals	71.6	8.0		75.5	9.7	
Frequency of vegetable or fruit consumption	Daily in at least twice a day	72.9	8.1	0.057	76.8	9.7	0.391
	Less often	72.1	8.5		74.7	13.7	

M, Mean; SD, Standard deviation; *p*, Statistical significance; T1, The first year of study; T2, The second year of study.

Regarding systolic blood pressure values, it was consistently noted that the average value was higher among students who consumed animal protein in the majority of their meals daily, with statistical significance observed in both years (*p* < 0.001 and *p* = 0.086, respectively). Additionally, there was a trend toward elevated systolic blood pressure among students reporting lower quality of life and decreased satisfaction with their health during the initial year of study, as well as those exhibiting higher alcohol consumption and reduced physical activity levels in the second year of studies.

Conversely, significantly elevated diastolic blood pressure values were observed among students who frequently consumed meals rich in animal protein during the first year of the study (*p* = 0.048). Furthermore, during the following year of observation, higher diastolic blood pressure was associated with students reporting satisfaction with their health (*p* = 0.024) and engaging in regular physical activity (*p* = 0.051).

Ultimately, higher BMI values were significantly correlated with increased systolic blood pressure values across the entire observation period, with *R* = 0.37 (*p* < 0.001) in the first year and *R* = 0.33 (*p* < 0.001) in the second year of the study. A similar relationship was documented for diastolic blood pressure, although obtained values of Spearman correlation coefficient were lower, *R* = 0.19 (*p* < 0.001) and *R* = 0.27 (*p* < 0.001), respectively in the first and second year of observation.

Table 5 presents the prevalence of overweight and obesity in both years of observation including selected explanatory variables. In the initial year of evaluation, 371 individuals (86.9%) exhibited normal body weight, 47 (11.0%) were overweight, and 9 (2.1%) were classified as obese. Subsequent assessments during the second year revealed the following distribution: 277 students (84.2%) with normal body weight, 40 (12.2%) classified as overweight, and 12 (3.6%) identified as obese. For 6 individuals at T2, missing data made it impossible to assess overweight and obesity. The calculated odds ratios highlight a significantly heightened incidence of overweight or obesity among first-year students who reported: male sex (OR = 2.809; 95% CI: 1.587–4.973); dissatisfaction with their health (OR = 2.145; 95% CI: 1.215–3.787); worse financial situation (OR = 2.307; 95% CI: 1.292–4.120); deteriorating health condition (OR = 2.738; 95% CI: 1.532–4.892); diagnosis of a chronic illness by a physician (OR = 1.855; 95% CI: 1.010–3.412); and consumption of animal protein-rich foods in at least 75% of daily meals (OR = 2.512; 95% CI: 1.326–4.761). Within the same cohort during their second year of study, a significantly elevated occurrence of overweight or obesity was associated with male sex (OR = 3.479; 95% CI: 1.891–6.398); dissatisfaction with health status (OR = 2.057; 95% CI: 1.128–3.759); worse financial situation (OR = 1.949; 95% CI: 1.045–3.634); and current cigarette smoking (OR = 2.197; 95% CI: 0.908–4.878), although this result lacks statistical significance; regular consumption of animal protein-rich foods (OR = 2.785; 95% CI: 1.400–5.555). In summary, regardless of the academic year, variables significantly associated with an increased risk of being overweight or obese include dissatisfaction with personal health, financial strain, and a diet abundant in animal products.

**Table 5:** The number and percentage of overweight or obese students in both observation years.

Explanatory variable		T1 (*N* = 427)		T2 (*N* = 329)	
		Overweight or obesity *N* = 56 (13.1%)	*p*-value	Overweight or obesity *N* = 52 (15.8%)	*p*-value
Sex	Male (*N*1 = 138; *N*2 = 108)	30 (21.7%)	<0.001	30 (27.8%)	<0.001
	Female (*N*1 = 289; *N*2 = 221)	26 (9.0%)		22 (9.9%)	
Declared quality of life	Average or poor (*N*1 = 117; *N*2 = 89)	18 (15.4%)	0.393	17 (19.1%)	0.318
	Good or very good (*N*1 = 310; *N*2 = 240)	38 (12.3%)		35 (14.5%)	
Satisfaction of health status	Yes, satisfied (*N*1 = 146; *N*2 = 118)	28 (9.9%)	0.007	17 (14.4%)	0.566
	Average or dissatisfied (*N*1 = 281; *N*2 = 208)	28 (19.2%)		35 (16.8%)	
Frequency of alcohol consumption	Never or rarely (*N*1 = 175; *N*2 = 298)	25 (14.3%)	0.560	48 (16.1%)	0.641
	More often than 2–4 times a week (*N*1 = 251; *N*2 = 31)	31 (12.3%)		4 (12.9%)	
Current financial situation	Average or poor (*N*1 = 115; *N*2 = 87)	24 (20.9%)	0.003	20 (23.0%)	0.033
	Good or very good (*N*1 = 312; *N*2 = 241)	32 (10.3%)		32 (13.3%)	
Current place of residence during studies at university	Family home (*N*1 = 124; *N*2 = 85)	17 (13.7%)	0.815	13 (15.3%)	0.869
	Dormitory/rented flat or room (*N*1 = 303; *N*2 = 243)	39 (12.9%)		39 (16.0%)	
Current cigarette smoking (traditional or electronic)	Never or in the past, not now (*N*1 = 358; *N*2 = 37)	45 (12.6%)	0.419	10 (27.0%)	0.048
	Yes, still (*N*1 = 68; *N*2 = 291)	11 (16.2%)		42 (14.4%)	
Frequency of physical activity	Yes, regularly or less often (*N*1 = 347; *N*2 = 260)	41 (11.8%)	0.088	42 (16.1%)	0.554
	Not at all (*N*1 = 79; *N*2 = 68)	15 (18.9%)		9 (13.2%)	
Current health status	Average or poor (*N*1 = 175; *N*2 = 129)	35 (20%)	<0.001	25 (19.4%)	0.153
	Good or very good (*N*1 = 251; *N*2 = 200)	21 (8.4%)		27 (13.5%)	
Ever diagnosed with chronic disease	Yes (*N*1 = 101; *N*2 = 93)	19 (18.8%)	0.044	17 (18.3%)	0.439
	No (*N*1 = 324; *N*2 = 236)	36 (11.1%)		35 (14.8%)	
Number of meals per day	At least 3 a day (*N*1 = 378; *N*2 = 285)	47 (12.4%)	0.247	44 (15.4%)	0.596
	Less than 3 (*N*1 = 49; *N*2 = 43)	9 (18.4%)		8 (18.6%)	
Number of meals containing animal protein	In all meals, at least 75% of meals (*N*1 = 244; *N*2 = 191)	42 (17.2%)	0.003	40 (20.9%)	0.002
	Fewer meals (*N*1 = 183; *N*2 = 138)	14 (7.6%)		12 (8.7%)	
Frequency of milk or cheese consumption	Daily in at least once a day (*N*1 = 271; *N*2 = 215)	36 (13.3%)	0.930	38 (17.7%)	0.201
	Less often (*N*1 = 154; *N*2 = 114)	20 (13.0%)		14 (12.3%)	
Frequency of vegetables or fruit consumption	Daily in at least twice a day (*N*1 = 283; *N*2 = 220)	32 (11.3%)	0.107	35 (15.9%)	0.941
	Less often (*N*1 = 142; *N*2 = 109)	24 (16.9%)		17 (15.6%)	
Frequency of raw vegetables or fruit consumption	Daily, at least 75% of meals (*N*1 = 193; *N*2 = 137)	24 (12.4%)	0.705	22 (16.1%)	0.931
	Less often (*N*1 = 234; *N*2 = 191)	32 (13.7%)		30 (15.7%)	
Frequency of wholemeal bread, groats, and dry leguminous vegetables consumption	Daily at least in one meal, at least 75% of meals (*N*1 = 273; *N*2 = 185)	32 (11.7%)	0.245	26 (14.0%)	0.323
	Less often (*N*1 = 153; *N*2 = 144)	24 (15.7%)		26 (18.1%)	

*N*, Number; *N*1, Number in the first year of study; *N*2, Number in the second year of study; *p*, Statistical significance; T1, The first year of study; T2, The second year of study.

## 4 Discussion

The main objective of our study was to evaluate the prevalence of overweight and obesity among medical students taking into account changes during a 2-year observation period and their selected determinants. Data for the general population demonstrated nearly 60% of adults in the WHO European Region are burdened with overweight or obesity, while almost one in three children contend with the same issue. Obesity represents a complex health problem, manifested in various determinants and consequences (23). The obtained results of our study revealed that 56 students (13.10%) in the first year of medical university were overweight or obese, and 52 (15.8%) in the second. Regardless of the academic year, the increased risk of being overweight or obese was significantly associated with dissatisfaction with individual health status, male sex, financial deficiencies, and a diet abundant in meat consumption. Studies conducted among student populations highlight geographic and cultural variations in the prevalence of obesity and overweight. For instance, research conducted among medical and healthcare faculties in Saudi Arabia and the United Arab Emirates revealed that 34.7–41.5% of participants were classified as overweight or obese (24–26), with 22.1% falling into the same category in Syria’s population of medical students (27). Furthermore, a study conducted among the extensive population of Western Balkans medical students indicated the prevalence of overweight was 12% and obesity was 2.3% (28). The findings obtained in the cited study are in line with our observations. Additionally, the results from the referenced studies confirmed that the male sex was a positive predictor of overweight and obesity, with current smoking status emerging as an additional significant factor.

In most cases, the reports regarding the prevalence of overweight and obesity among medical students are elaborated on a cross-sectional research approach. In contrast, our study allows us to identify the changes in these health concerns over time. The education phase of university students is considered a time when a young person has an opportunity to test himself in an independent life, and thus make his own choices and bear their consequences, which directly impact the quality of life. Medical studies, due to the high level and amount of knowledge to be acquired in a short time, often require students to focus mainly on education at the expense of chronic stress, lack of activity, reaching for stimulants such as alcohol or cigarettes, and incorrect eating habits. Co-occurrence of these factors may influence the prevalence of weight disorders leading to overweight or obesity during studies and adult life. BMI is a useful anthropometric indicator for screening assessment of body composition as well as nutritional status. In terms of BMI calculation, in our study, a significant increase in score has been identified during the second year of observation. In a prospective observational Deliens et al. study, concerning changes in body weight and body composition across 5 years at university, an increase in both BMI and body fat was found in approximately 70% of students, with a statistically significantly higher increase in men than in women, in contrary to the results of own study. What is more, the highest increase in BMI and fat occurred during the first semester and in the last year of studies (29). Similar results were obtained in a 3-year study of health faculty students, which showed an average weight gain of 1.6 kg in men. The reason for these results could be found in a statistically significant increase in diet energy intake and a decrease in physical activity in students after the first year of their studies (3). In our study, higher BMI was observed in medical students who consumed animal protein in their everyday diet, which can be explained by the higher content of high-calorie saturated fats in their meals. Moreover, throughout the entire observation period, a relationship has been observed between higher BMI levels and increased consumption of a diet abundant in meat products. Interestingly, the assessment of dietary patterns as poor and the percentage of daily meat consumption was slightly higher in the second year of the study compared to the first. Additionally, in the second year of observation, a significant proportion of male participants and students reporting a poor financial situation were identified among those who were overweight or obese, contrasting with the previous year. We hypothesize that the heightened prevalence of these factors in the second year of observation may have contributed to an increased prevalence of overweight and obesity over the 2 years. Available published data also describes the relationship between overweight and obesity, and emotional eating. For instance, factors such as prolonged psychological distress, anxiety, or depression may provoke unhealthy dietary patterns and are matched with an increase in body weight (30). While not directly addressed in our study, it is plausible that emotional eating contributes to the increased frequency of overweight and obesity observed in the second year of our study.

However, underweight and obesity are considered age-related issues as well. Scientific research indicates that progressive mitochondrial dysfunction plays a key role in the development of overweight and obesity. The reasons for the dynamic impairment of mitochondrial function in young people can be found in an unhealthy diet, rich in processed products that are a source of saturated fatty acids and salt. Medical students in the second year of the study more often than in the first year declared that their diet was insufficient (“poor diet assessment”). They also admitted that they less often consumed wholemeal bread, groats, and dry leguminous vegetables in their daily diet, unlike alcohol consumption. The aging of the body is associated with a physiological slowdown of metabolism and, consequently, an increase in abdominal obesity. Both, early mitochondrial dysfunction and adipocyte hyperplasia, which also leads to metabolic dysregulation and may be caused by an incorrect lifestyle in students, support the physiological process of slowing down the metabolism. It may clarify higher BMI values in students in the second year of our study in comparison to the first year (31–33).

Traditional or electronic cigarette smoking is a high-powered addiction. People start to experiment with smoking in late childhood or adolescence and they continue smoking while studying. Some research showed that cigarette smoking could decrease blood pressure (BP), as a result of a temporary reduction in the smoker’s stress level. Medical students are the future authorities in the field of health care and for this reason, society expects, they should lead a healthy lifestyle. The presented study reported that 16% of examined students used to smoke during the first year of observation and the number of smokers decreased to 11% in the second year of observation. Students who smoked cigarettes represented lower systolic blood pressure values than non-smokers in both, the first and second years of observation. On the other hand, the study demonstrated a statistically significant positive correlation between BMI and both, systolic and diastolic blood pressure values in the examined group of students. Some studies indicate cardiometabolic disorders in supposedly healthy nonobese young people, which could be caused by chronic cigarette smoking, alcohol consumption, and poor dietary habits (34–36).

In conclusion, the determinants of overweight and obesity are complex and difficult to explain unequivocally. The factors contributing to overweight and obesity among medical students may be similar to those affecting the general population but can also include issues specific to this group. On the one hand, a study conducted among a large group of fourth-year medical students identified the primary causes of overweight and obesity as physical inactivity, overeating, marketing of unhealthy food, poor nutritional knowledge, psychological problems, genetic factors, and metabolic defects such as endocrine disorders (37). Additionally, students’ irregular schedules and daily lifestyles can make it challenging to maintain a healthy diet. Notably, the consumption of sweet beverages is relatively high among students, which is associated with an increased risk of obesity (38). A common factor among students is certain overall unhealthy lifestyle behaviors that coexist and interact, thereby increasing the risk of overweight and obesity in this population (39).

### 4.1 Strengths and limitations

According to the Supreme Audit Office’s recent report, 24% of adults in Poland are obese and most of the people with excessive body weight were recorded in the Silesian Voivodeship—69.2% of the population (40). Our findings indicate that the prevalence of overweight or obesity among medical students aligns more closely with data from the European Balkan region than with that of Arab countries, in terms of both geographical and cultural Background. The study we conducted addresses a timely and enduring topic of universal relevance. By adhering to strict inclusion criteria, we achieved a participation rate of over 80% among first- and second-year medical students, effectively encompassing nearly the entire available population and ensuring a representative sample. It is also one of the few studies that show the change or maintenance of determinants influencing the lifestyle, body weight, and nutritional status of young people over time because, in our study, medical students were surveyed twice in the 2-year interval. Our findings underscore the significance of early-life quality of life on the development of body weight disorders and associated health implications later in life. Specifically, we identify a relationship between unsatisfactory quality of life among students and the potential for future overweight or obesity-related health issues. The attention of local and university government authorities should be drawn to help students shape healthy attitudes also through systemic activities, such as nutritional education, access to nutritious food in campus canteens, and cooperation with dieticians. Moreover, among the solutions aimed at enhancing students’ well-being, Mindfulness-Based Interventions (MBIs) have proven to be particularly effective (41). Programs that include meditation classes, dietary guidance, and brief yoga sessions have been shown to reduce overall distress significantly. Additionally, these interventions improve attention maintenance, resilience, mental well-being, and emotional regulation (42). In a broader context, early interventions that help medical students manage stress and develop healthy nutrition habits can include the continuous promotion of physical activity and sleep hygiene (43). An intriguing solution for medical students, as part of the younger population, could be the development and promotion of mobile apps and online platforms. These tools could offer resources on healthy eating, exercise routines, and mental health support.

The primary limitations of the study revolve around its relatively small sample size, which consists solely of students from the Medical University of Silesia in Katowice. However, it is worth noting the high participation rates observed during both data collection periods. Furthermore, the prospective nature of the study lends credibility to the significance of the findings presented. Furthermore, our ongoing prospective study of medical students includes plans for a third observation point (T3), which is scheduled to take place during their fourth year of studies.

## 5 Conclusion

1. The results of our study confirmed an increase in the prevalence of overweight or obesity among medical students during the 2-year follow-up observation.
2. Dissatisfaction with individual health status, male sex, financial deficiencies, and a diet abundant in meat consumption were significant determinants of overweight or obesity among medical students.
3. Our findings indicate the necessity for medical schools to actively encourage a healthy lifestyle within their curriculum and incorporate programs aimed at fostering positive behaviors among medical students.

## Data Availability

The raw data supporting the conclusions of this article will be made available by the authors, without undue reservation.

## References

[1] Olszanecka-Glinianowicz M Mazur A Chudek J Kos-Kudła B Markuszewski L Dudek D. Obesity in adults: position statement of polish Association for the Study on obesity, polish Association of Endocrinology, polish Association of Cardiodiabetology, polish psychiatric association, section of metabolic and bariatric surgery of the Association of Polish Surgeons, and the College of Family Physicians in Poland. Nutrients. (2023) 15:1–41. doi: 10.3390/NU15071641 PMC10097178 37049479

[2] Vadeboncoeur C Foster C Townsend N. Freshman 15 in England: a longitudinal evaluation of first year university student’s weight change. BMC Obes. (2016) 3:1–9. doi: 10.1186/s40608-016-0125-1 27826452 PMC5095959

[3] Czeczelewski J Czeczelewski M Czeczelewska E. Longitudinal weight and body-composition changes in polish undergraduate students of health faculty. J Am Coll Heal. (2023) 71:1084–90. doi: 10.1080/07448481.2021.1920604, PMID: 34242519

[4] de Vos P Hanck C Neisingh M Prak D Groen H Faas MM. Weight gain in freshman college students and perceived health. Prev Med Rep. (2015) 2:229–34. doi: 10.1016/J.PMEDR.2015.03.008, PMID: 26844076 PMC4721347

[5] Haraldstad K Wahl A Andenæs R Andersen JR Andersen MH Beisland E. A systematic review of quality of life research in medicine and health sciences. Qual Life Res. (2019) 28:2641–50. doi: 10.1007/S11136-019-02214-9, PMID: 31187410 PMC6761255

[6] Panzini RG Mosqueiro BP Zimpel RR Bandeira DR Rocha NS Fleck MP. Quality-of-life and spirituality. Int Rev Psychiatry. (2017) 29:263–82. doi: 10.1080/09540261.2017.1285553 28587554

[7] Cai T Verze P Bjerklund Johansen TE. The quality of life definition: where are we going? Urology. (2021) 1:14–22. doi: 10.3390/uro1010003

[8] Ribeiro ÍJS Pereira R Freire IV de Oliveira BG Casotti CA Boery EN. Stress and quality of life among university students: a systematic literature review. Health Profess Educ. (2018) 4:70–7. doi: 10.1016/j.hpe.2017.03.002

[9] de Vasconcelos HCA Fragoso LVC Marinho NBP de Araújo MFM de Freitas RWJF Zanetti ML. Correlation between anthropometric indicators and sleep quality among brazilian university students. Rev Esc Enferm USP. (2013) 47:852–9. doi: 10.1590/S0080-623420130000400012 24310682

[10] Henning M Krägeloh C Hawken S Zhao Y Doherty I. Quality of life and motivation to learn: A study of medical students. Issues Educ Res. (2010) 12:437–45. doi: 10.1007/s12564-011-9148-y

[11] Ismail NAH Tekke M. Rediscovering Rogers’s self theory and personality. J Educ Health Comm Psychol. (2015) 4:2088–3129.

[12] Gajewska D Harton A. Current nutritional status of the polish population—focus on body weight status. J Health Inequal. (2023) 9:154–60. doi: 10.5114/jhi.2023.133899

[13] Gajda M Sedlaczek K Szemik S Kowalska M. Determinants of alcohol consumption among medical students: results from POLLEK cohort study. Int J Environ Res Public Health. (2021) 18:5872. doi: 10.3390/IJERPH18115872 34070755 PMC8199068

[14] Desaimd MN Millermd WC Terrillbravendermd B. Risk factors associated with overweight and obesity in college students. Coll Health. (2008) 57:109–14. doi: 10.3200/JACH.57.1.109-114 18682353

[15] Jamali A Tofangchiha S Jamali R Nedjat S Jan D Narimani A. Medical students’ health-related quality of life: roles of social and behavioural factors. Med Educ. (2013) 47:1001–12. doi: 10.1111/MEDU.12247 24016170

[16] Szemik S Gajda M Gładyś A Kowalska M. The association between COVID-19 pandemic and the quality of life of medical students in Silesian Voivodeship, Poland. Int J Environ Res Public Health. (2022) 19:1–14. doi: 10.3390/ijerph191911888, PMID: 36231191 PMC9565595

[17] Barański K Szemik S Kaleta-Pilarska A Kowalska M. General health and its relation to the quality of life and alcohol consumption in a polish cohort of medical students—preliminary results of POLLEK survey. Front Public Health. (2023) 11:1178124. doi: 10.3389/fpubh.2023.1178124 37469698 PMC10352118

[18] World Health Organization (WHO). Obesity and overweight. (2024). Available online at: https://www.who.int/news-room/fact-sheets/detail/obesity-and-overweight (Accessed April 15, 2024).

[19] Centers for Disease Control and Prevention (CDC). High Blood Pressure. (2024). Available online at: https://www.cdc.gov/bloodpressure/about.htm (Accessed April 15, 2024).

[20] Kowalska M Skrzypek M Danso F Humeniuk M. Assessment of reliability of the WHOQOL-BREF questionnaire in a study of quality of life among adults, the economically active population of the Silesian agglomeration. Zdrow Publ. (2012) 66:531–7. 23230727

[21] Nowicki GJ Polak M Ślusarska B Czernecki K. The relationship between diet and the occurrence of depressive symptoms in a community example with high rates of social deprivation: a cross-sectional study. Nutrients. (2023) 15:1–15. doi: 10.3390/nu15173778, PMID: 37686809 PMC10489963

[22] Mazurek-Kusiak AK Kobyłka A Korcz N Sosnowska M. Analysis of eating habits and body composition of young adult poles. Nutrients. (2021) 13:1–19. doi: 10.3390/nu13114083, PMID: 34836335 PMC8624486

[23] Vandevijvere S De Pauw R Djojosoeparto S Gorasso V Guariguata L Løvhaug AL. Upstream determinants of overweight and obesity in Europe. Curr Obes Rep. (2023) 12:417–28. doi: 10.1007/s13679-023-00524-1, PMID: 37594616

[24] Makkawy E Alrakha A Al-Mubarak A Alotaibi H Alotaibi N Alasmari A. Prevalence of overweight and obesity and their associated factors among health sciences college students, Saudi Arabia. J Family Med Prim Care. (2021) 10:961. doi: 10.4103/jfmpc.jfmpc_1749_20 34041105 PMC8138400

[25] Mahfouz AA Alsaleem SA Alsaleem MA Ghazy RM. Prevalence of obesity and associated dietary habits among medical students at King Khalid University, Southwestern Saudi Arabia. Medicina. (2024) 60:347. doi: 10.3390/medicina60030347, PMID: 38541073 PMC10972436

[26] El-Kader RGA Ogale RJ Zidan OO Al Jadaan O Kumardhas V Ahmed SK. Assessment of health-related behaviors among medical students: a cross-sectional study. Health Sci Rep. (2023) 6:e1310. doi: 10.1002/hsr2.1310, PMID: 37292103 PMC10244612

[27] Alhashemi M Mayo W Alshaghel MM Brimo Alsaman MZ Haj KL. Prevalence of obesity and its association with fast-food consumption and physical activity: a cross-sectional study and review of medical students’ obesity rate. Ann Med Surg. (2022) 79:1–7. doi: 10.1016/j.amsu.2022.104007 PMC9289307 35860087

[28] Ilić M Pang H Vlaški T Grujičić M Novaković B. Prevalence and associated factors of overweight and obesity among medical students from the Western Balkans (South-East Europe region). BMC Public Health. (2024) 24:29. doi: 10.1186/s12889-023-17389-7, PMID: 38166959 PMC10763029

[29] Deliens T Deforche B Chapelle L Clarys P. Changes in weight and body composition across five years at university: a prospective observational study. PLoS One. (2019) 14:e0225187. doi: 10.1371/journal.pone.0225187, PMID: 31721793 PMC6853334

[30] Dakanalis A Mentzelou M Papadopoulou SK Papandreou D Spanoudaki M Vasios GK. The association of emotional eating with overweight/obesity, depression, anxiety/stress, and dietary patterns: a review of the current clinical evidence. Nutrients. (2023) 15:1–18. doi: 10.3390/nu15051173, PMID: 36904172 PMC10005347

[31] López-Lluch G. Mitochondrial activity and dynamics changes regarding metabolism in ageing and obesity. Mech Ageing Dev. (2017) 162:108–21. doi: 10.1016/j.mad.2016.12.005 27993601

[32] Jura M Kozak LP. Obesity and related consequences to ageing. Age. (2016) 38:23. doi: 10.1007/s11357-016-9884-3, PMID: 26846415 PMC5005878

[33] Moldakozhayev A Gladyshev VN. Metabolism, homeostasis, and aging. Trends Endocrinol Metab. (2023) 34:158–69. doi: 10.1016/j.tem.2023.01.003, PMID: 36681595 PMC11096277

[34] Jena S Purohit K. Smoking status and its effect on blood pressure: a study on medical students. CHRISMED J Health Res. (2017) 4:14. doi: 10.4103/2348-3334.196034

[35] Williams RA Rose AM Bruno RS Hanks AS Kennel JA McDonald JD. Examination of the relationship of diet quality with cardiometabolic risk factors in apparently healthy college students. J Educ Health Promot. (2019) 8:148. doi: 10.4103/jehp.jehp_12_19, PMID: 31544113 PMC6745876

[36] Balgoon MJ Al-Zahrani MH Alkhattabi NA Alzahrani NA. The correlation between obesity and metabolic syndrome in young female university students in the Kingdom of Saudi Arabia. Diabet Metab Syndr. (2019) 13:2399–402. doi: 10.1016/j.dsx.2019.06.015, PMID: 31405650

[37] Phelan SM Burgess DJ Burke SE Przedworski JM Dovidio JF Hardeman R. Beliefs about the causes of obesity in a national sample of 4th year medical students. Patient Educ Couns. (2015) 98:1446–9. doi: 10.1016/j.pec.2015.06.017, PMID: 26143579 PMC4747081

[38] Durán-Agüero S Valdés-Badilla P Valladares M Espinoza V Mena F Oñate G. Consumption of ultra-processed food and its association with obesity in Chilean university students: a multi-center study: ultra-processed food and obesity in Chilean university students. J Am Coll Heal. (2023) 71:2356–62. doi: 10.1080/07448481.2021.1967960 34469253

[39] Telleria-Aramburu N Arroyo-Izaga M. Risk factors of overweight/obesity-related lifestyles in university students: results from the EHU12/24 study. Br J Nutr. (2022) 127:914–26. doi: 10.1017/S0007114521001483, PMID: 33955337 PMC8908003

[40] Supreme Audit Office (2023). Obesity prevention and treatment overwhelmed the system. 1–87. Available online at: https://www.nik.gov.pl/plik/id,28874,vp,31705.pdf (Accessed May 16, 2024).

[41] Hathaisaard C Wannarit K Pattanaseri K. Mindfulness-based interventions reducing and preventing stress and burnout in medical students: a systematic review and meta-analysis. Asian J Psychiatr. (2022) 69:102997. doi: 10.1016/j.ajp.2021.102997, PMID: 34995839

[42] Fazia T Bubbico F Nova A Buizza C Cela H Iozzi D. Improving stress management, anxiety, and mental well-being in medical students through an online mindfulness-based intervention: a randomized study. Sci Rep. (2023) 13:8214. doi: 10.1038/s41598-023-35483-z, PMID: 37217666 PMC10201046

[43] Boyd A Mealand K Briggs Early K Oestreich E. Perceived stress, grit, and self-care behaviors in first-year medical students. Am J Lifestyle Med. (2023) 17:803–12. doi: 10.1177/15598276221124576 38511119 PMC10948928

